# Formation of magnetic nanowire arrays by cooperative lateral growth

**DOI:** 10.1126/sciadv.abk0180

**Published:** 2022-01-28

**Authors:** Fei Chen, Zihao Yang, Jing-Ning Li, Fei Jia, Fan Wang, Di Zhao, Ru-Wen Peng, Mu Wang

**Affiliations:** 1 National Laboratory of Solid State Microstructures, School of Physics, and Collaborative Innovation Center of Advanced Microstructures, Nanjing University, Nanjing 210093, China.; 2 American Physical Society, Ridge, NY 11961, USA.

## Abstract

Nanowires typically grow along their longitudinal axis, and the long-range order among wires sustains only when a template exists. Here, we report an unprecedented electrochemical growth of ordered metallic nanowire arrays from an ultrathin electrolyte layer, which is achieved by solidifying the electrolyte solution below the freezing temperature. The thickness of the electrodeposit is instantaneously tunable by the applied electric pulses, leading to parallel ridges on webbed film without using any template. An array of metallic nanowires with desired separation and width determined by the applied pulses is formed on the substrate with arbitrary surface patterns by etching away the webbed film thereafter. This work demonstrates a previously unrecognized fabrication strategy that bridges the gap of top-down lithography and bottom-up self-organization in making ordered metallic nanowire arrays over a large area with low cost.

## Teaser

We present a method of growing ordered nanowire arrays, bridging the gap of top-down lithography and bottom-up self-organization.

## INTRODUCTION

Fabrication of nanostructures can be categorized into top-down and bottom-up approaches (*1*, *2*). The top-down approach uses various kinds of lithography, which has unprecedented accuracy and controllability, yet requires sophisticated equipment and expensive operating costs (*3*, *4*). On the other hand, the bottom-up approaches are cost-efficient and do not rely on sophisticated facilities (*5*–*7*). However, it is determined by self-organization, which lacks strict repeatability and spatial homogeneity over a long distance. Is there any way to bridge the gap between top-down lithography and bottom-up self-organization? So far, many efforts have been devoted to solve this issue. Template such as porous anodic aluminum oxide (AAO) contains high-density self-organized uniform pore arrays and has been applied to fabricate regular patterns (*8*–*10*). The diameter of the pores is of the scale of tens of nanometers, and the length reaches a few hundreds of micrometers. Regular metallic (*8*) or alloy (*9*) nanowires can be electrochemically fabricated via the nanochannels of AAO. However, those nanowire arrays can rarely be free-standing, and the long-range order among the nanowires vanishes once the template is removed (*10*). With a different approach, the micronozzle can move in three-dimensional (3D) space and release ions at specific locations to initiate electrochemical growth (*11*, *12*). Yet, the fabrication efficiency depends on the scanning speed of the nozzle, and the fabrication accuracy remains challenging as well.

For decades, electrodeposition in a thin layer of electrolyte sandwiched by two insulating substrates has been used to study the basic principles of pattern formation (*13*–*15*). To suppress electroconvections (*15*–*17*), we introduce an ultrathin layer of the electrolyte by freezing the electrolyte to form a concentrated ultrathin electrolyte layer between the electrolyte ice and the substrate (*18*–*20*). As a result, the branching rate of the metal deposition reduces significantly, and compact homogeneous metallic thin film and periodic structures are formed (*18*–*20*). However, the exact growth mechanism in the ultrathin layer remains unclear so far.

Here, we demonstrate an unusual electrochemical fabrication of a metallic nanowire array around the freezing temperature of the electrolyte solution. We apply the electric voltage pulses across two straight, parallel electrodes. Hence, the thickness of the electrodeposit film is instantaneously modulated by the electric pulses, forming straight, parallel ridges separated by webbed films. The metallic nanowires with desired spatial separation and line width are obtained by gently etching away the webbed film thereafter. The separation and width/height of the nanowires depend on the frequency and shape of the applied pulse. Unlike conventional nanowires growing along their longitudinal axial direction (*21*–*23*), here the whole nanowire simultaneously develops in the direction perpendicular to the wire axis. Besides, the nanowires can be fabricated on a surface with any topography, including silicon grating with a high–aspect ratio surface pattern.

## RESULTS

**Fig. 1.**
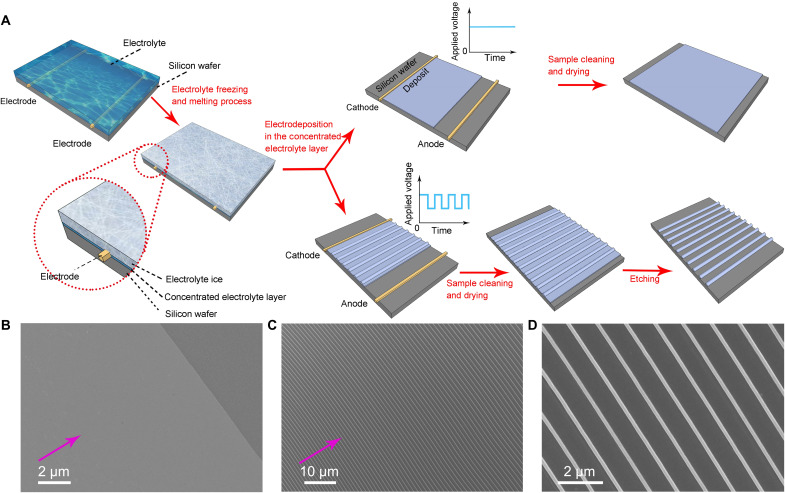
Lateral growth enables smooth and patterned thin film growth. (**A**) Schematics of the fabrication process for the high-quality cobalt thin film and nanowire array. (**B**) The flat film deposited by applying a constant voltage in electrodeposition. (**C**) The nanowire array of cobalt generated by using a voltage with static component 1.1 V plus a square-shaped oscillatory component with amplitude 0.3 V and frequency 0.2 Hz. The magenta-colored arrow denotes the growth direction of the electrodeposit. (**D**) The details of the wire array shown in (C).

The experiments are carried out with an aqueous solution of CoSO_4_ (0.03 M) sandwiched by two rigid boundaries with two straight electrodes, as illustrated in Fig. 1A. To generate the ultrathin electrolyte layer, we carefully freeze the sandwiched electrolyte solution to single-crystalline ice by decreasing temperature. Eventually, the system temperature is set as −1.8°C, 1.7°C below the freezing point of the 0.03 M CoSO_4_ aqueous solution. In the freezing process, due to the partitioning effect, CoSO_4_ is partially expelled from the ice, forming an ultrathin, highly concentrated electrolyte layer between ice and substrate when the equilibrium is reached. Our electrochemical growth takes place in this ultrathin layer (*18*–*20*). From the temperature of the experiment, we estimate that the concentration in the ultrathin thin layer is about 1.6 M (the freezing point of the CoSO_4_ electrolyte decreases as the concentration increases, see fig. S1). Meanwhile, a homogeneous cobalt thin film is formed if a constant voltage is applied, as shown in Fig. 1B. The growth direction of the metallic film is in-plane of the substrate and is perpendicular to the electrodes, as illustrated by the magenta arrow. The root-mean-square roughness of the film is less than 1 nm across an area of 1 μm^2^ (see fig. S2). By applying a square electric pulse superimposed on a constant voltage, periodic ridges separated by webbed films are obtained (Fig. 1C). Thereafter, the webbed film (~50 nm in thickness) between the ridges can be easily removed by gentle chemical etching. In this way, a well-defined cobalt nanowire array is formed on the substrate, as illustrated in Fig. 1D.

**Fig. 2.**
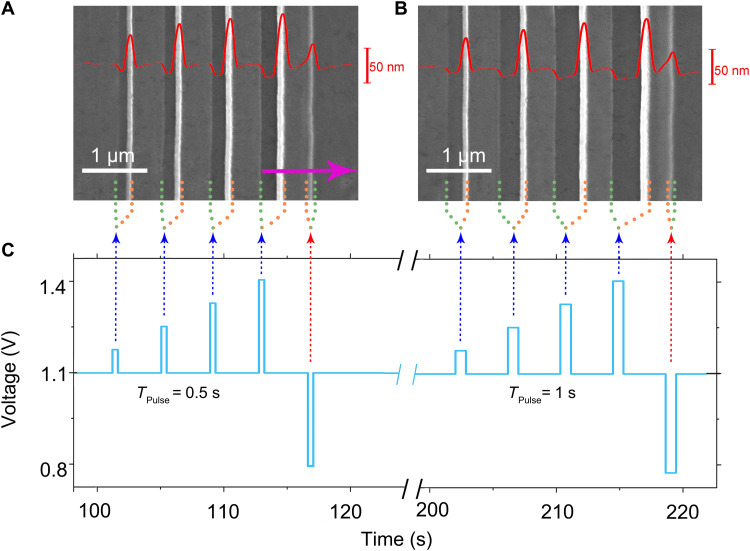
The structures fabricated by the programed pulsed signal. (**A** and **B**) The SEM micrographs of the electrodeposit grown by different pulsed signals. The magenta arrow denotes the growth direction of the electrodeposit. On each frame, the superimposed red curve is the height profile measured by AFM. (**C**) The programmed pulsed signal is applied in electrodeposition. The dotted blue and red arrows correlate the positive and negative pulse signals and growth patterns, respectively. The positive pulse amplitude increases linearly from 0.075 to 0.3 V, and the pulse duration is 0.5 s for (A) and 1.0 s for (B).

To understand this growth phenomenon, we apply a series of positive and negative electric voltage pulses with different amplitudes in the electrodeposition. The topography of the generated electrodeposit is illustrated in Fig. 2 (A and B), where the red curves demonstrate the topography along the line on the deposited film measured by atomic force microscopy (AFM). Each ridge in Fig. 2 (A and B) corresponds to the pulse shown in Fig. 2C, as marked by the dashed arrows. For the positive pulses, the voltage jumps from the constant value (1.1 V) to the selected higher voltage, sustains for 0.5 and 1.0 s, respectively (Fig. 2C), and then drops back to the constant voltage. At the constant voltage, the electrodeposit extends as a homogeneous film, corresponding to the gray strips on the left side of each green dashed line in Fig. 2 (A and B). Corresponding to the rising edge of the positive pulse, the voltage suddenly jumps from 1.1 V to a higher value. This voltage jump boosts the nucleation rate and accelerates the interface advance rate. Meanwhile, the film becomes much thinner than that formed at 1.1 V. Consequently, a strip with darker contrast appears, and it sustains when the applied voltage remains on the top of the electric pulse. From Fig. 2 (A and B), one may find that the width of the dark strip is proportional to the height and the duration of each pulse. At the falling edge of the pulse, the voltage drops suddenly. Accordingly, the nucleation rate decreases sharply, yet those already-formed Co nuclei continue to grow. As a result, a ridge of cobalt crystallites builds up, and it continues to grow until a new balance of nucleation and film extension is reached at 1.1 V. The ridges are shown as the bright white lines beside the yellow dashed lines in Fig. 2 (A and B). The homogeneous webbed film (gray strip) grows until the next pulse arrives. Figure 2 (A and B) also shows that as the amplitude of the pulse increases, not only does the width of the dark strip increase, the shade of the strip also becomes darker, suggesting that the film becomes thinner. The height of the ridges depends on the amplitude of the pulses as well, which is confirmed by both the contrast of the scanning electron microscopy (SEM) micrography and the AFM profiles of the ridges shown in Fig. 2 (A and B).

We also introduce negative pulses, where the voltage decreases lower than the constant voltage 1.1 V, as marked by the red dashed arrows in Fig. 2. A ridge still forms corresponding to the falling edge of the pulse, but with a much lower height than that created by the positive pulses.

**Fig. 3.**
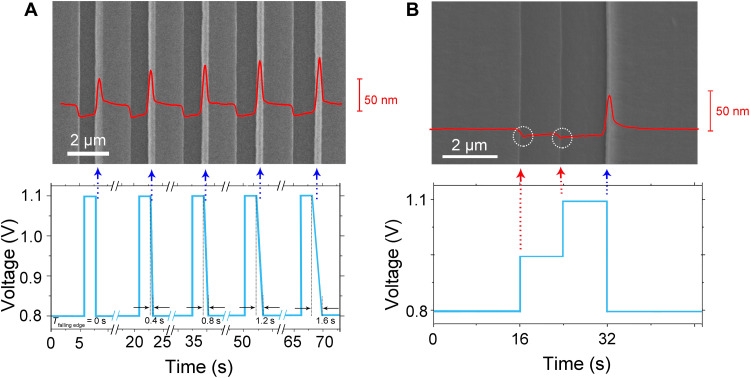
Dependence of the size of nanowire on the programmed voltage signals. (**A**) The SEM micrograph of the electrodeposit generated by the programmed voltage signal with AFM profile of the wire array. The pulse duration is 2 s, and the pulse amplitude is 0.3 V. The time of the descending edge of the pulse varies from 0 to 1.6 s. Correspondingly, the thickness of the wire increases, as indicated by the AFM profile (red). (**B**) The SEM image of the thin film deposited by the signal below. The superimposed red curve is the AFM profile of the electrodeposit. When the voltage jumps higher, the contrast of the cobalt film becomes darker, suggesting that the thickness of the film becomes relatively thinner. No ridges appear corresponding to the voltage jump-up, as marked by the red arrows.

Figure 3A demonstrates the influence of the shape of the applied pulse on the formation of ridges. By increasing the duration of the falling edges from 0 to 1.6 s while keeping the amplitudes of the pulses as 0.3 V, the ridges on the webbed film become higher accordingly (Fig. 3A). As illustrated in Fig. 3B, a pulse with two rising edges and one falling edge has been applied. When the voltage increases as steps, the thickness of the film decreases accordingly, which is confirmed by both the contrast of the SEM and AFM micrographs (Fig. 3B). No ridges appear corresponding to the voltage jump-up, as marked by the red arrows in Fig. 3B. However, a distinct ridge (about 40 nm in height) emerges at the falling edge, as indicated by the blue arrow in Fig. 3B. Therefore, we confirm that the ridges occur only at the falling edges of the pulses. By modulating the width and the shape of the applied pulse, nanowires with desired size and separation can be fabricated.

**Fig. 4.**
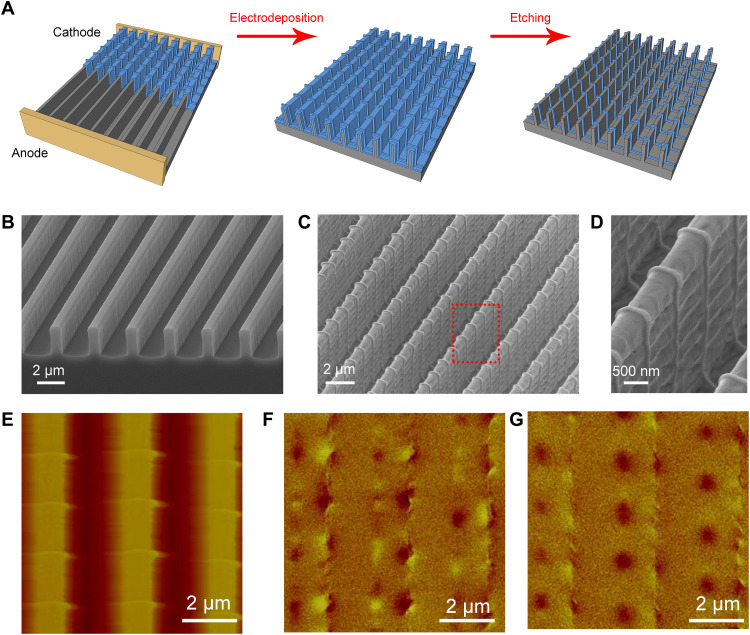
Formation of the standing U-shaped structure by electrodeposition. (**A**) The schematics of the fabrication process of the standing U-shaped nanowire arrays on a substrate fabricated by deep silicon etching. (**B**) The SEM micrograph of the silicon deep-etched substrate. The width of each stripe is 1 μm, and the depth of the ditch is 3 μm. The period of the trenches is 3 μm. (**C**) The micrograph of the cobalt electrodeposits grown on the patterned substrate. A square-shaped voltage is applied in electrodeposition. (**D**) The enlarged details of the electrodeposit developed on the silicon grating. (**E**) Topographic micrograph of the nanowire arrays measured by AFM. (**F**) The original magnetization state of the nanowire arrays after electrodeposition and etching. (**G**) The remanence magnetization state of the nanowire arrays after being magnetized by a magnetic field of 0.3 T.

The growth described above can occur on a surface with any topography. We carry out electrodeposition on a silicon grating with a high–aspect ratio surface topography (Fig. 4A). The details of the substrate are illustrated in Fig. 4B. Straight electrodes are set perpendicular to the trenches. By applying periodic square pulses, parallel ridges are generated, which follow the surface topography of the silicon grating and form a stereo standing U-shaped magnetic nanowire array (Fig. 4C). Last, an array of separated standing U-shaped nanowires is fabricated by etching away the webbed film between the neighboring ridges (Fig. 4D).

We carry out electron diffraction of the electrodeposited film and wires, as marked by the red-dashed circles in figs. S7 and S8. The dotted diffraction rings indicate that the deposit is polycrystalline. Hence, the magnetocrystalline anisotropy of the nanowire is negligible compared to its shape anisotropy. Because of the shape anisotropy of nanowire, the easy magnetization direction of a wire is along its long axis. Magnetic force microscopy (MFM) is applied to detect the stray magnetic field of the stereo magnetic nanowire array. One may identify head-to-head and tail-to-tail domain walls on the nanowires on the top surface of the trenches (Fig. 4F), which may be used for 3D magnetic storage (*24*–*26*). However, the as-grown magnetic domain state is not necessarily the ground state of the system. When the nanowire arrays are magnetized with a magnetic field of 0.3 T, which is 45° to the plane of the substrate and perpendicular to the trenches of the substrate, the tail-to-tail domain walls are regularly pinned at one side of the corners of the nanowire arrays (Fig. 4G). We also notice in the experiment that the nonuniform local magnetic field of the cantilever of MFM may move the magnetic domains. As indicated by the dashed ellipse in fig. S4, a tail-to-tail domain wall in a 50-nm-high nanowire can be continuously pushed along the nanowire in MFM measurement, whereas in thicker nanowires, the magnetic domains cannot be moved easily. By pushing a tail-to-tail domain wall toward a nearby head-to-head domain wall, annihilation of head-to-head and tail-to-tail domain wall pairs has been observed (dashed rectangle in fig. S5).

## DISCUSSION

The formation of ridges at the falling edge of the applied voltage pulse is a universal phenomenon. It does not depend on the shape of the electric pulses. As illustrated in fig. S3, triangular negative pulses with different falling times and amplitudes are applied. The height of the ridge increases with the falling times and amplitudes accordingly.

Two effects are essential for the unique growth phenomena presented here. One is that nucleation of metal crystallite occurs preferentially at the concave corner of the substrate and the metal deposit, which guarantees the metal deposits to develop laterally on the surface of the substrate. This effect is responsible for the lateral growth of electrodeposits on the substrate, leading to many other patterns with long-range order (*27*, *28*). The other is the freezing of electrolyte solution behind the growing front in electrodeposition. It is known that the electrolyte concentration behind the growth front becomes essentially zero in electrodeposition (*13*, *15*). It follows that the liquid behind the growth front solidifies at a temperature below the freezing point. In this way, the electrodeposit is immediately embedded by ice after the growth. In other words, deposition occurs only on the very front tip of the growing interface. This feature keeps the boundary of the ridge and the webbed film very sharp and helps coordinate the growth of the webbed film and ridges.

Now we can understand this unique nanowire growth as follows. The lateral growth stabilizes when the extension of the electrodeposit and the cation transport from the anode reaches an equilibrium. The nucleation and growth of the electrodeposit consume cations and deplete local electrolyte concentration behind the growth front. Therefore, the grown electrodeposit is embedded by ice at the temperature below the freezing point, and only the very front of the electrodeposit can grow. Corresponding to the rising edge of the positive pulse, nucleation is suddenly accelerated, and the electrodeposit moves forward faster. Meanwhile, the lateral grown film becomes thinner, forming a dark strip aside of the gray film. When the applied voltage drops, the nucleation rate decreases suddenly, so interfacial lateral extension decelerates substantially. Meanwhile, cations that have been driven by the earlier higher voltage accumulate at the growth front. The existing nuclei at the growth front hence grow larger and form a more elevated ridge. After that, once the cation transportation from the anode becomes compatible with the film growth at the temperature, stabilized film growth restarts at the lower constant voltage. By repeating this process, a pattern with long-range order can be obtained. It is noteworthy that this fabrication process can also be applied to grow nanowire arrays of other metals, such as copper and nickel-iron alloy (fig. S6).

It should be pointed out that the interfacial growth and the cation transfer are controlled by the Laplacian fields, which are self-organized processes. In our unique system, the interfacial growth, cation transfer, and electrolyte solidification are modulated by the applied electric pulses and controlled macroscopically. In this sense, it is beyond conventional self-organization. It is an organized growth controlled by external electric pulses. We suggest that the electrochemical growth of stereo metallic nanowire arrays presented here could be promising to solve the tremendous challenges in fabricating the microstructures for racetrack memory, the high-performance, solid-state, nonvolatile memory device using a spin-polarized electric current to drive magnetic domains along magnetic nanowires, and hence realizing much smaller physical device dimensions than comparable solid-state memory devices (*25*). This stereo nanowire growth avoids complicated lithography techniques, has easily tunable interwire separation, and eliminates the irregularity and randomness in conventional self-organization.

To summarize, we demonstrate in this report a unique electrodeposition approach to fabricate stereo metallic nanowire arrays, with the geometrical parameters of the array controlled by both the applied voltage pulses and substrate topography. The applied pulses help coordinate the growth of the thin webbing film and the nanoridges, while freezing the electrolyte and the corner-mediated lateral growth guarantee the smoothness of the electrodeposit and the sharp response of the growth morphology to the external electric pulses. This previously unexpected growth mode bridges the gap between top-down lithography and bottom-up self-organization, demonstrating a convenient and economical way to fabricate functional stereo microstructures.

## MATERIALS AND METHODS

Sample fabrication is carried out in an electrodeposition cell below the freezing temperature of the electrolyte solution. The electrolyte solution is prepared by dissolving analytical reagent CoSO_4_ (Alfa Aesar, 99.5%) in deionized water (Millipore; electric resistivity: 18.2 megohm·cm). The initial concentration of CoSO_4_ aqueous electrolyte is 0.03 M. Forty microliters of electrolyte solution is sandwiched by two rigid boundaries made of a microscope cover glass and a polished silicon wafer. Two parallel, straight cobalt wires (0.1 mm in diameter, Alfa Aesar, 99.995%) are used as the cathode and anode. The horizontal separation of the electrodes is fixed as 6 mm. This electrodeposition cell is placed in a chamber made of copper. The temperature of the chamber is controlled by a thermostat circulator (temperature range: −20° to 100°C, accuracy: 0.01°C, Cole-Parmer, Polystat 12108-35). The details of the chamber design can be found in (*18*, *29*). A Peltier element (size: 23 mm by 23 mm by 3.7 mm, maximum power: 14.7 W) is placed underneath the electrodeposition cell to stimulate nucleation in solidifying the electrolyte. A thermocouple monitors the local temperature near the silicon wafer. An optical microscope (Leitz, Orthoplan-pol) is used to observe the nucleation and growth of the electrolyte ice and the electrodeposition process.

In the experiment, the temperature of the circulating refrigerant (50% glycol and 50% deionized water) in the thermostat circulator is set as −1°C. Because of the system’s heat loss, the temperature of the thermal chamber eventually stabilizes at about −0.1°C when the room temperature is 20°C. An electric current is applied to the Peltier element to stimulate nucleation of ice of electrolyte. By carefully controlling the electric current applied on the Peltier element, nucleation and melting of nuclei can be controlled. By repeating this process, we keep only one nucleus of the electrolyte ice in the view field of the microscope. After that, the chamber temperature is slowly decreased (typical decreasing rate: −0.1°C/hour) to the targeting temperature, and the ice nucleus grows stably. Eventually, a flat, uniform single-crystalline ice of electrolyte is generated. When the equilibrium is reached, an ultrathin electrolyte is trapped between the ice and the substrates because of the partitioning effect in the solidification process. We carry out the electrodeposition in this ultrathin electrolyte layer. The whole growing process is monitored with an optical microscope.

We use a signal generator (Sony, AFG320) to apply a specific voltage pattern across the electrodes. The applied voltage varies from a constant voltage or a constant voltage superposing with a pulsed voltage. The shape and frequency of the pulses can be programmed. Usually, the electrodeposit nucleates on a silicon or glass surface. The electrodeposit grows from the cathode toward the anode in the ultrathin electrolyte between the ice and the substrates. After the electrodeposition, the sample is rinsed with deionized water and dried in a vacuum chamber for further analysis. To remove the webbed film between the nanowires, we apply Ar^+^ ion beam etching with the etching rate of the cobalt element calibrated in advance (Gatan, PECS682, beam current: 300 μA).

To fabricate the stereo standing U-shaped nanowires, we introduce silicon grating with a high–aspect ratio surface topography as the substrate for electrodeposition. The silicon grating is manufactured by the Bosch gas-switching technique (*30*). We first prefabricate the photoresist (Allresist, AR-N 7520.17, thickness: 500 nm) pattern on the silicon wafer with photolithography. Then, the sample is isotropically etched by reactive ions generated by CF_4_ and SF_6_ gases. Meanwhile, C_4_F_8_ is introduced to provide fluorocarbon as polymer coating of the entire surface of the silicon as a passivation layer. The fluorocarbon layer coated on the horizontal surfaces is immediately sputtered away by the vertical collision of the ions. However, the fluorocarbon layer coated on the sidewall survives physical etching and can only be slowly etched by the chemical reaction. The manifestation of sidewall roughness is a direct consequence of the alternating etching and sidewall passivation cycles.

With the silicon grating as the substrate, the electrodeposition process is carried out to fabricate the standing U-shaped nanowires. The webbed film between the stereo nanowires can hardly be etched by the Ar^+^ beam since the webbed film on the sidewall and that at the bottom of the silicon trenches cannot be efficiently removed by the ion beams. So, we carry out chemical etching for the stereo nanowires. To realize homogeneous etching rates, we oxidized the metallic deposit slightly at 200°C for 10 min in an atmosphere of 100% oxygen in a quartz tube furnace. Then, the sample was etched in 0.5 mM H_2_SO_4_ aqueous solution for 30 min. Last, the standing U-shaped nanowire array on the silicon grating is rinsed with deionized water and dried in a vacuum chamber for further characterization.

The morphology of cobalt nanowires is characterized by a field-emission scanning electron microscope (Zeiss, ULTRA 55) and multimode AFM in tapping mode (Digital Instruments, Nanoscope IIIa). The magnetic state of the nanowire array is characterized by the MFM mode with a lift height of 40 nm. The magnetization of the nanowires is performed by homemade Helmholtz coils, which generate a maximum magnetic field of 0.6 T.

## Supplementary Materials

### This PDF file includes:

Supplementary text Figs. S1 to S8 References
