# Supplementary material for: Formation of magnetic nanowire arrays by cooperative lateral growth
Source: Sci Adv. 2022 Jan 28;8(4):eabk0180. doi: 10.1126/sciadv.abk0180 (PMC8797794; doi:10.1126/sciadv.abk0180)
Supplement: Supplementary file 1 — Supplementary text Figs. S1 to S8 References [file sciadv.abk0180_sm.pdf]

Science Advances
AAAS

Supplementary Materials for

Formation of magnetic nanowire arrays by cooperative lateral growth

Fei Chen, Zihao Yang, Jing-Ning Li, Fei Jia, Fan Wang, Di Zhao, Ru-Wen Peng*, Mu Wang*

*Corresponding author. Email: muwang@nju.edu.cn (M.W.); rwpeng@nju.edu.cn (R.-W.P.)

Published 28 January 2022, *Sci. Adv.* **8**, eabk0180 (2022)
DOI: 10.1126/sciadv.abk0180

This PDF file includes:

Supplementary text
Figs. S1 to S8
References

Supplementary Text

Partitioning Effect in Solidifying the Electrolyte Film

When the electrolyte temperature decreases below -0.1°C, which is the freezing point of an electrolyte of 0.03 M, the nucleus of the electrolyte ice is induced and starts to grow. Due to the partitioning effect, $CoSO_4$ is partially expelled from the electrolyte ice into the liquid in solidification. The partitioning effect is known in crystallization: by solidifying electrolyte solution, the electrolyte (salt) is expelled from the ice, increasing the ion concentration in front of solid and liquid electrolyte interface when the partition coefficient of the salt is less than 1. In our specific system, $CoSO_4$ is expelled from the ice. When the single crystalline ice occupies the whole electro-deposition cell, the ultrathin layer of the highly concentrated electrolyte layer is trapped between ice and substrate. The freezing point of $CoSO_4$ electrolyte decreases when the concentration of $CoSO_4$ increases, as illustrated in fig. S1. When the equilibrium is reached at -1.8 °C, the trapped ultrathin electrolyte layer between the ice and the substrate will have a concentration around 1.6M.

The electrodeposition is carried out at -1.8 °C in this ultrathin electrolyte layer. On the other hand, it has been well established that local electrolyte concentration decreases due to the growth of metallic deposits in the electrodeposition process. Both theory [13] and experiments [15,31] show that the electrolyte concentration behind the growth front becomes essentially zero. Therefore, the liquid behind the growth front solidifies in the electrodeposition process. In other words, the electrodeposit is immediately embedded by ice when it is generated. Thus, the ice acts as a protector preventing further growth when the electrodeposit is formed.

Estimating the Thickness of the Ultrathin Layer of Electrolyte

The thickness of the ultrathin layer of the electrolyte can be estimated as follows. Consider the one-dimensional situation. A nucleus is initiated in the middle of the electrolyte and develops towards the upper and lower substrates. At the ice-liquid interface, due to the partitioning effect, the salt concentration follows

$$C_{\mathrm{i}} = k_0 \cdot C_l \,, \qquad (1)$$

where C_i is the salt concentration inside the electrolyte ice, and C_l is the salt concentration in the liquid electrolyte. k_0 is the partition coefficient of $CoSO_4$ in water. We assume that it is a constant. During the solidification of the electrolyte, the mass conservation of the salt requires

$$H \cdot C_0 = 2\int_0^s C_i ds + 2h \cdot C_l \,, \qquad (2)$$

where H is the initial thickness of the aqueous solution of $CoSO_4$; C_0 is the initial salt concentration in the electrolyte; s is the half-thickness of the ice film; h is the thickness of the trapped ultrathin

electrolyte layer. During the freezing process of the electrolyte, the ice thickness increases, and the thickness of the trapped electrolyte layer decreases. Yet the following relation remains

$$H = 2(h + s). \quad (3)$$

By taking eqs. (1) and (3) to eq. (2), one may obtain

$$H \cdot C_0 = 2k_0 \int_0^s C_l ds + (H - 2s) \cdot C_l. \quad (4)$$

Take the derivative of both sides of eq. (4) with respect to s, one may obtain

$$\frac{dC_l}{ds} + \frac{2(k_0 - 1)}{H - 2s} \cdot C_l = 0. \quad (5)$$

It follows that

$$\begin{aligned} C_l &= C \cdot e^{-\int \frac{2(k_0-1)}{H-2s} ds} \\ &= C \cdot (H - 2s)^{k_0 - 1}, \end{aligned} \quad (6)$$

where C is a constant to be determined by the boundary conditions. The initial salt concentration of the liquid electrolyte satisfies

$$C_l(s = 0) = C_0. \quad (7)$$

Take the boundary condition (7) to eq. (6), one may obtain

$$C = \frac{C_0}{H^{k_0 - 1}}; \quad (8)$$

$$C_l = C_0 \cdot \left(\frac{H - 2s}{H}\right)^{k_0 - 1}. \quad (9)$$

In our specific situation, the initial salt concentration in the electrolyte C_0 is 0.03 mol/L; the salt concentration in the ultrathin electrolyte after freezing process C_l is around 1.6 mol/L; the initial thickness of the aqueous solution of $CoSO_4$ is about 0.1 mm; the partition coefficient of $CoSO_4$ in water k_0 is about 0.1. From these data, we may estimate that the thickness of the ultrathin layer of electrolyte before electrodeposition is about 500 nm.

Lateral Growth of Flat Thin Film

We place anode and cathode separately on the surface of the substrate. The direction of the initial electrical field is dominated by the arrangement of electrodes, pointing from the cathode to the anode. By applying a constant voltage across the electrodes, the homogeneous thin film is formed laterally on the substrate surface, moving towards the anode. The growth rate is about 10 μm/min when the applied voltage is around 1.1 V. This scenario occurs since the electrodeposit prefers to nucleate at the concave corner of the substrate and the already grown metal deposit [19, 27]. It should be pointed out that this electrodeposit layer can be very flat. As illustrated below, the root-mean-square roughness of the electrodeposited film is about 0.958 nm across an area of one square micrometer (fig. S2).

According to the theory of electrochemical growth, when the growth front moves from the cathode towards the anode, the concentration behind the growth front will approach zero [13,15]. It follows that in our system, once the deposit is grown, the liquid after the growth front will be frozen, and the electrodeposit will be buried by the ice. This explains that the surface of the thin film can be so flat.

Tuning the Height of Cobalt Ridges by Designing the Shape of the Applied Electric Pulses

As we demonstrate in Fig. 2 of the main text, the ridges are generated at the falling edges of the applied pulses. The formation of ridges at the falling edge is a universal phenomenon and does not depend on the shape of the electric pulses. As illustrated in fig. S3, triangular negative pulses with different falling times and amplitudes are applied. One may find that when the descending edge extends from 0.5s to 8s, the ridge height increases from 25 nm to 100 nm (so do the width of the wire). We also introduce a series of pulses with the same descending time but different voltage amplitude, as illustrated in Fig. R10(B). When the falling edge of voltage increases from 0.067 V to 0.4 V, the ridge height increases from 5 nm to 35 nm accordingly (so does the width of the wires). The red curves on the SEM micrographs are the AFM topography profile of the ridges.

The Magnetic Domain Wall Motion Driven by MFM Tip

The geometrical shape of a magnetic nanowire usually defines its magnetization direction. The easy magnetization direction is in the long axis of the nanowires. The magnetic domain wall is a boundary across which each individual magnetic moment reorients. Each domain has a head (positive or north pole) and a tail (negative or south pole) in the nanowire. The domain walls in the nanowires alternate between head-to-head and tail-to-tail configurations and the stray magnetic fields can be detected as bright or dark spots by MFM [24]. We fabricated an array of cobalt nanowires of different sizes. The topography and the MFM micrographs are illustrated in figs. S4A and S4B. One may observe several bright and dark spots on the magnetic wires thinner than 112 nm, corresponding to head-to-head and tail-to-tail magnetic domain walls. The stray magnetic field of the domain walls is stronger for the thicker nanowires. However, for those wires wider than 120 nm, the easy magnetization becomes no longer along the long axis of the nanowires. The magnetic domain pattern turns more complicated. Interestingly, a prolonged dark spot in the thinnest nanowire can be observed, as indicated by the green dashed ellipse in fig. S4B. Usually,

the size of a domain wall is comparable to the width of the nanowire [24,32,33]. The prolonged dark spot does not reflect the actual magnetic state of the nanowire. As will be demonstrated later, this prolonged dark spot stands for the trace of the magnetic domain wall that has been pushed by the tip of the magnetic cantilever.

When a magnetic field of 0.6 T is applied parallel to the long axis of the nanowires and is gradually reduced to zero, the magnetic polarization of the nanowire aligns with the long axis of the wire. No domain walls are observed at the remanence state (fig. S4C). However, when a magnetic field of 0.6 T is applied perpendicular to the long axis of the nanowires and is gradually reduced to zero, head-to-head and tail-to-tail magnetic domain walls emerge randomly, as shown in fig. S4D.

To investigate especially the prolonged dark spot in fig. S4B, we fabricate a nanowire array with a width less than 50 nm. We repeatedly capture the MFM micrograph in the cantilever's downward and upward scanning, as shown in fig. S5. The topography of the nanowires is illustrated in fig. S5A. The figs. S5B, S5C, and S5D demonstrate the MFM micrographs of the same region captured in upward and downward scans, respectively. We focus on the magnetic state inside of the blue dashed rectangular frames. For example, in fig. S5B, we can find a pair of bright and dark spots corresponding to a pair of head-to-head and tail-to-tail domain walls in the blue dashed frame. Meanwhile, the cantilever scans downwards. When the cantilever scans upwards, as illustrated in fig. S5C, the dark spot at the bottom of the blue dashed frame has been pushed upwards by the tip of the cantilever, forming an elongated dark spot. Then, near the top of the frame, the dark spot stops moving upwards further, and the previous bright spot disappears as well. This is confirmed by fig. S5D (downward scan), in the same blue dashed frame, both the dark and the bright spots disappear. This observation suggests that annihilation of a pair of head-to-head and tail-to-tail domain walls in the upward scanning process in fig. S5C occurs, which is pushed by the MFM cantilever. After the annihilation, the dashed blue frame region becomes a single magnetic domain free of any domain wall. This observation indicates that the local magnetic state can be changed in MFM scanning.

This phenomenon indicates that the coercivity of thin nanowires is low. The magnetic domain of the thin nanowires thus can be affected by the moving magnetic cantilever. The magnetic tip we use here is purchased from Nano Sensor, PPP-MFMR. This effect can be eliminated by using a low-moment MFM probe instead of a standard MFM probe.

The Structural analysis of the Deposited Film and Etched Nanowires

With our current growth conditions, the cobalt film is polycrystalline. The electron diffraction pattern of the film is shown in fig. S7, where the dotted rings can be identified. We have also carried out the TEM analysis of the crystallographic structure of the nanowires, as shown in fig. S8. The electron diffraction pattern of the selected area indicates that the nanowire is polycrystalline as well. As a result, the easy magnetization direction of the cobalt nanowires is determined by the shape anisotropy of the nanowires, which is along the long axis of the nanowires.

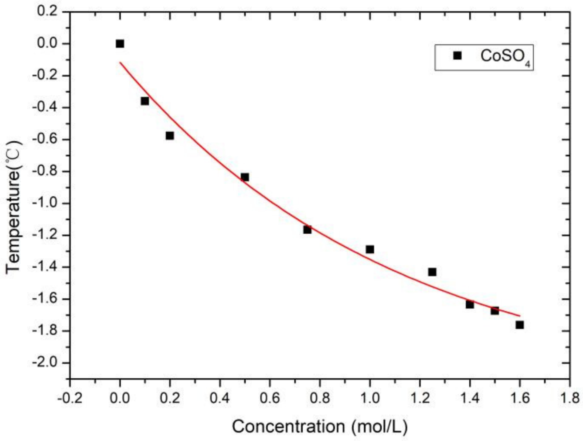

Fig. S1. The dependence of the freezing point of $CoSO_4$ electrolyte and the concentration. The dots are measured in experiments at ambient pressure.

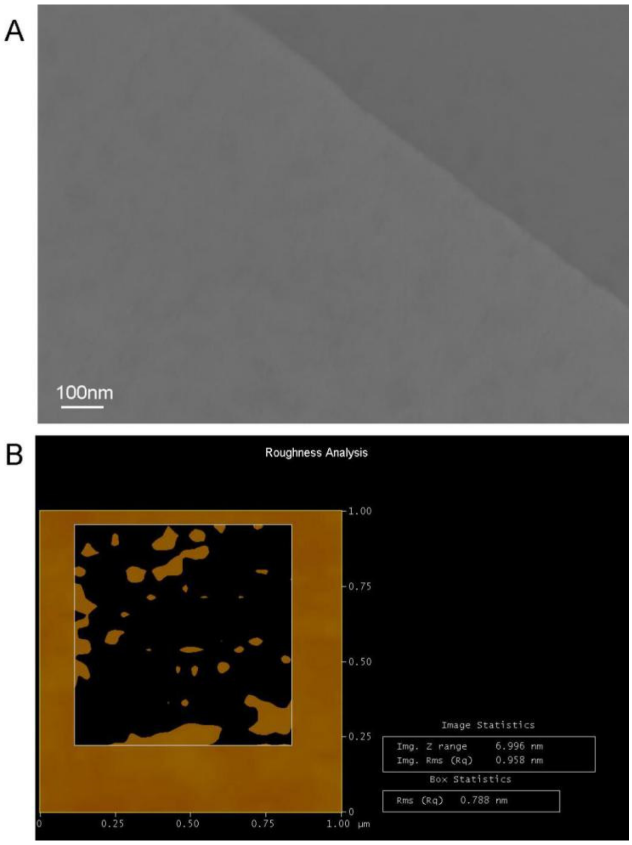

Fig. S2. The smooth electrodeposited thin film characterized by SEM and AFM. (A)The SEM micrograph of the thin film. (B) The roughness analysis of the electrodeposited thin film.

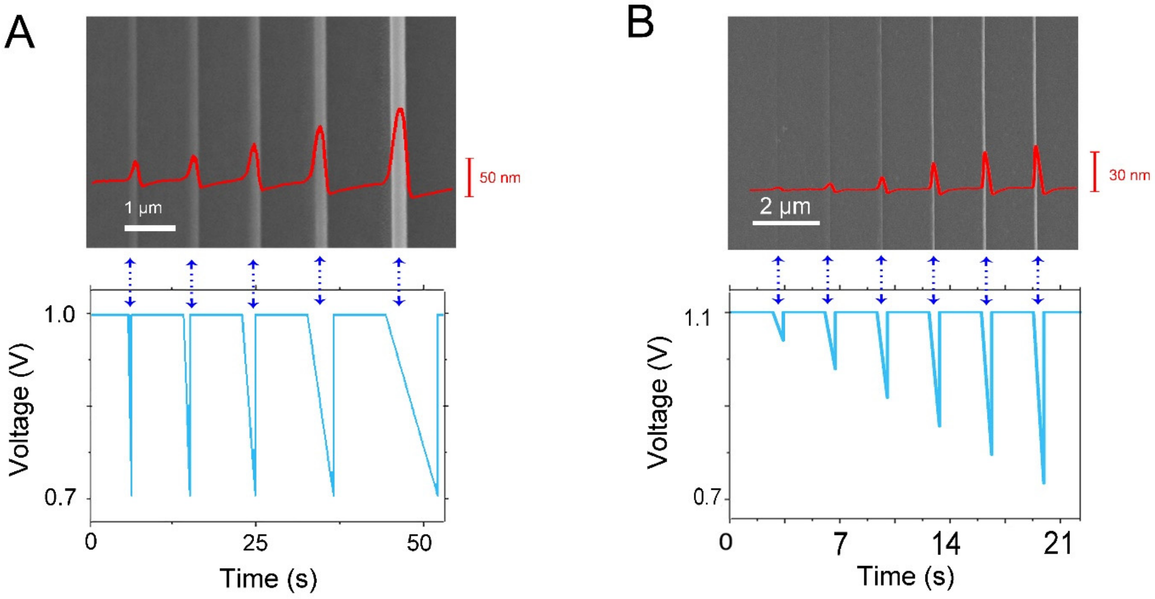

Fig. S3. The nanowire arrays fabricated by the programed triangular-shaped voltage signals. (A) The SEM image of the thin film deposited by the triangular-shaped voltage with different falling times. The red curve is the height profile measured by AFM. The growth direction of the film is from left to right. The duration of the triangular pulses is 0.5s, 1s, 2s, 4s, and 8s, respectively. The amplitude of the pulse signals is kept the same as 0.3 V. The thickness of the thin film is larger if the duration of the pulse signal is longer. (B) The SEM image of the thin film deposited by the triangular-shaped pulses with different amplitudes. The designed amplitudes of the voltage pulse are linearly decreasing from 0.067 V to 0.4 V. The heights of nanowires increase with the amplitudes of the pulse.

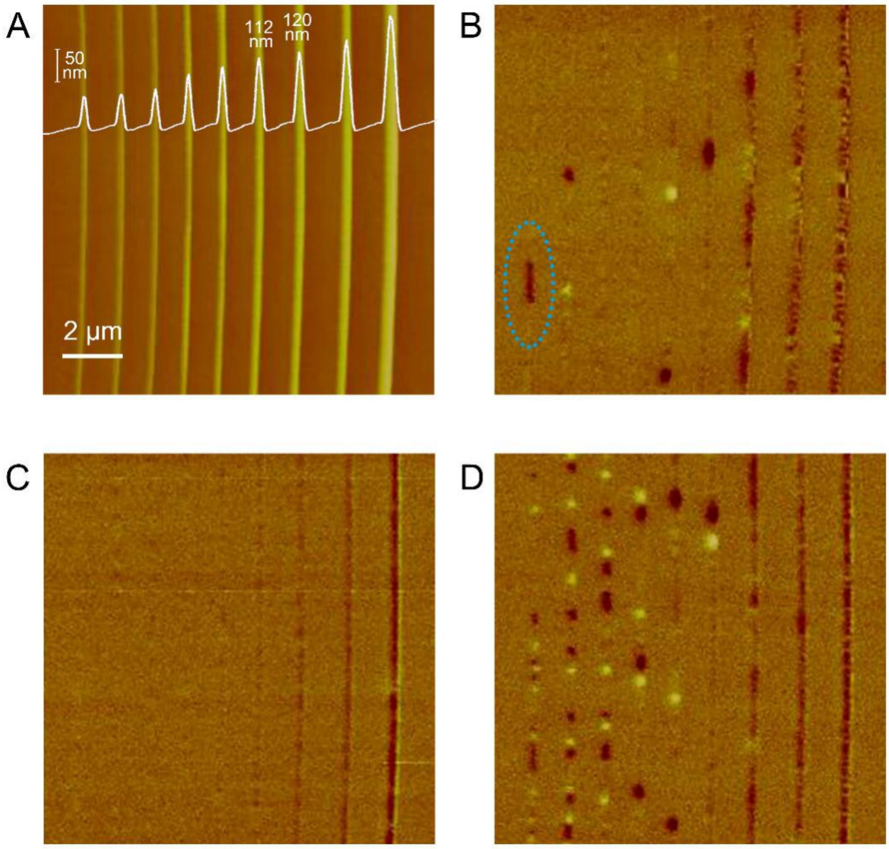

Fig. S4. MFM images of nanowires with increasing height and width. (A) The topography of the nanowires. (B) The initial magnetic state of the nanowires. The magnetic signal captured in the dashed green ellipse is the trace of the tail-to-tail domain wall motion in MFM imaging. (C) The remanence state of the nanowires after a magnetic field of 0.6T is applied parallel to the nanowires. (D) The remanence state of the nanowires after a magnetic field of 0.6T is applied perpendicular to the plane of the nanowire array.

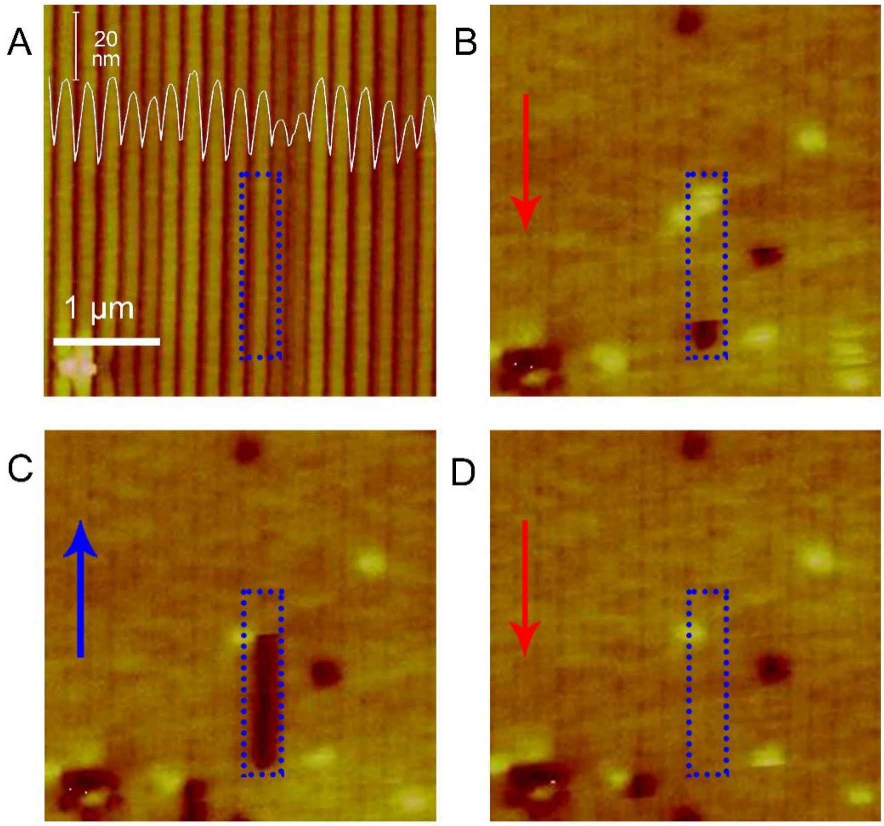

Fig. S5. MFM images of the trace of domain wall motion and annihilation. (A) The topography of the nanowires. (B) The initial magnetic state of the nanowires. A pair of head-to-head and tail-to-tail domain walls appear in the dashed blue rectangle. The cantilever moves downwards when this micrograph is captured. (C) The magnetic state of the nanowires when the cantilever scans upwards. The tail-to-tail domain wall is driven upwards by the tip, forming an elongated dark spot. Close to the position of the previous bright spot, the dark spot stops extending, and the earlier bright spot disappears as well, suggesting the occurrence of the annihilation of the domain walls. (D) The magnetic state of the nanowires when the cantilever scans downward again. Both the head-to-head and tail-to-tail domain walls on the nanowire vanish in the dashed rectangle.

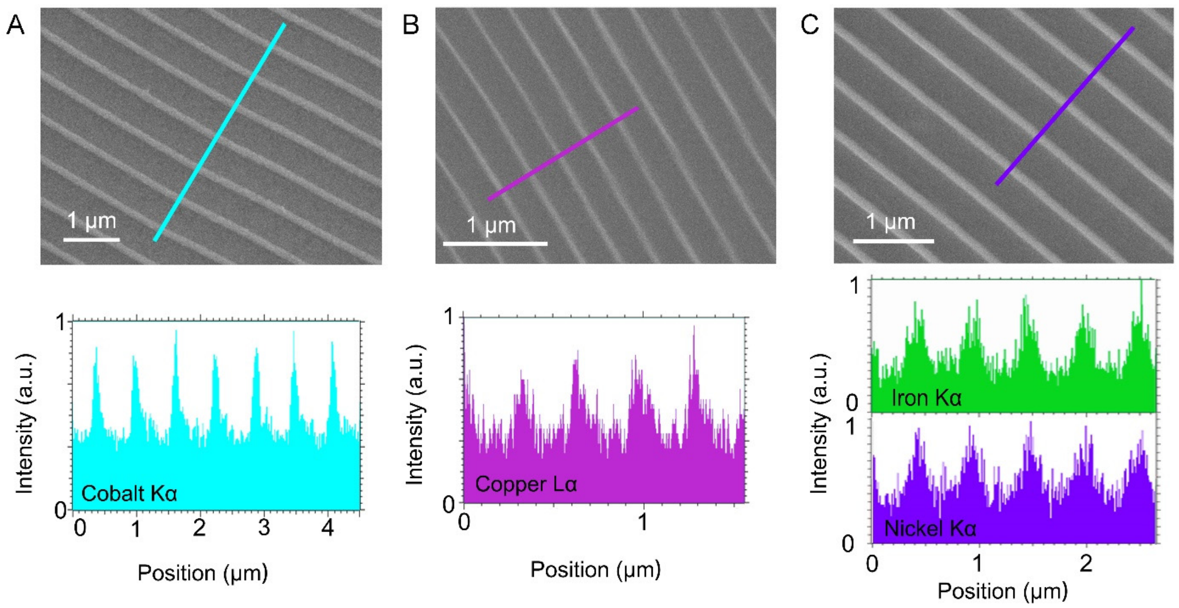

Fig. S6. The fabricated different metallic and alloy nanowire arrays and their elemental analysis. SEM micrographs of the cobalt (A), copper (B), and NiFe alloy (C) nanowires and the corresponding energy-dispersive X-ray spectroscopy data measured along the colored lines.

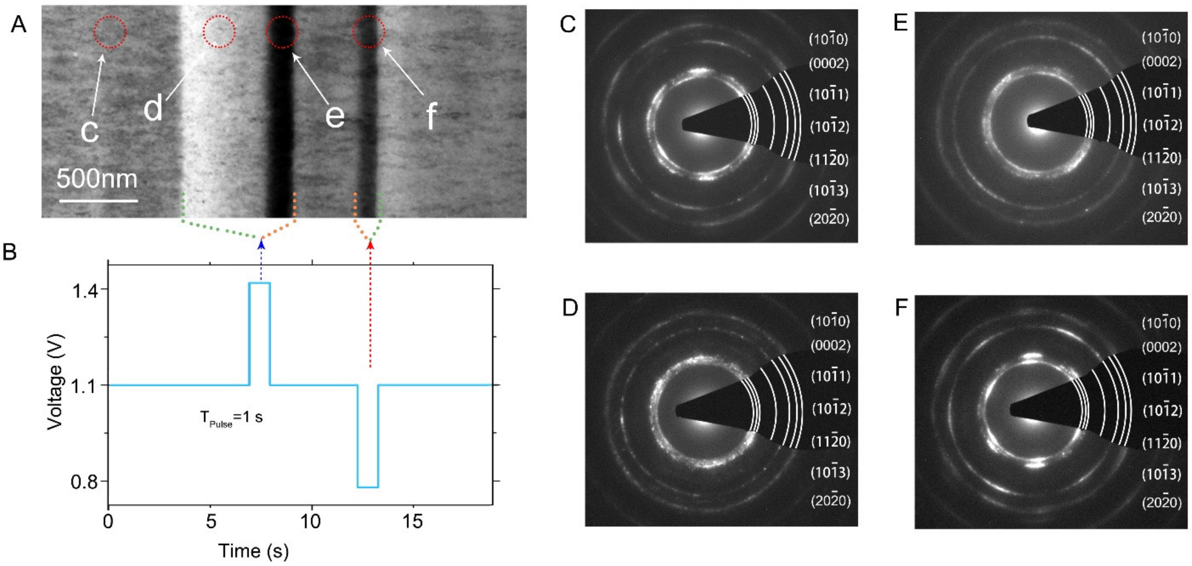

Fig. S7. The structural analysis of the electrodeposited film fabricated in different applied voltage. TEM micrograph of a cobalt film(A) electrodeposited by the voltage signal in (B) and its electron diffraction patterns(C-F) for the selected sites (dashed red circles marked as ***c***-***f***) in (A). Site ***c*** corresponds to the thin film deposited at 1.1V. The site ***d*** corresponds to a much thinner film generated the higher voltage at 1.4V of the first pulse in (B). Site ***e*** in (A) corresponds to the ridge of cobalt, which is formed at the falling edge of the pulse, dropping from 1.4V to 1.1V. Site ***f*** in (A) represents the second ridge of cobalt, which is formed during the falling edge of the pulse from 1.1V to 0.8V.

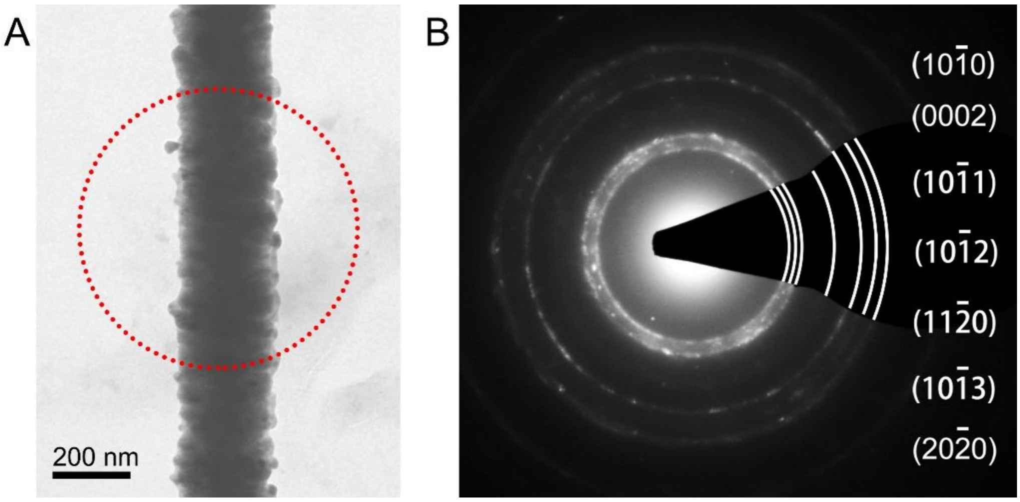

Fig. S8. The structural analysis of the nanowire. TEM micrograph of a cobalt nanowire(A) and its electron diffraction pattern (B) for the selected site (dashed red circle) in (A). This result indicates that the nanowire is polycrystalline.

REFERENCES AND NOTES

1. B. P. Isaacoff, K. A. Brown, Progress in top-down control of bottom-up assembly. *Nano Lett.* **17**, 6508–6510 (2017)

2. S. Gantenbein, K. Masania, W. Woigk, J. P. W. Sesseg, T. A. Tervoort, A. R. Studart, Three-dimensional printing of hierarchical liquid-crystal-polymer structures. *Nature* **561**, 226–230 (2018)

3. M. Hentschel, M. Schäferling, X. Duan, H. Giessen, N. Liu, Chiral plasmonics. *Sci. Adv.* **3**, e1602735 (2017)

4. V. K. Sangwan, M. C. Hersam, Neuromorphic nanoelectronic materials. *Nat. Nanotechnol.* **15**, 517–528 (2020)

5. C. Moreno, M. Vilas-Varela, B. Kretz, A. Garcia-Lekue, M. V. Costache, M. Paradinas, M. Panighel, G. Ceballos, S. O. Valenzuela, D. Peña, A. Mugarza, Bottom-up synthesis of multifunctional nanoporous graphene. *Science* **360**, 199–203 (2018)

6. P. Li, N. A. Vermeulen, C. D. Malliakas, D. A. Gómez-Gualdrón, A. J. Howarth, B. L. Mehdi, A. Dohnalkova, N. D. Browning, M. O'Keeffe, O. K. Farha, Bottom-up construction of a superstructure in a porous uranium-organic crystal. *Science* **356**, 624–627 (2017)

7. D. X. Luong, K. V. Bets, W. A. Algozeeb, M. G. Stanford, C. Kittrell, W. Chen, R. V. Salvatierra, M. Ren, E. A. McHugh, P. A. Advincula, Z. Wang, M. Bhatt, H. Guo, V. Mancevski, R. Shahsavari, B. I. Yakobson, J. M. Tour, Gram-scale bottom-up flash graphene synthesis. *Nature* **577**, 647–651 (2020)

8. K. R. Hebert, S. P. Albu, I. Paramasivam, P. Schmuki, Morphological instability leading to formation of porous anodic oxide films. *Nat. Mater.* **11**, 162–166 (2011)

9. Z. Zhang, X.-Y. Kong, K. Xiao, G. Xie, Q. Liu, Y. Tian, H. Zhang, J. Ma, L. Wen, L. Jiang, A bioinspired multifunctional heterogeneous membrane with ultrahigh ionic rectification and highly efficient selective ionic gating. *Adv. Mater.* **28**, 144–150 (2016)

10. B. Guo, L. Tian, W. Xie, A. Batool, G. Xie, Q. Xiang, S. U. Jan, R. Boddula, J. R. Gong, Vertically aligned porous organic semiconductor nanorod array photoanodes for efficient charge utilization. *Nano Lett.* **18**, 5954–5960 (2018)

11. Y.-G. Park, H. S. An, J.-Y. Kim, J.-U. Park, High-resolution, reconfigurable printing of liquid metals with three-dimensional structures. *Sci. Adv.* **5**, eaaw2844 (2019)

12. J. H. Je, J.-M. Kim, J. Jaworski, Progression in the fountain pen approach: From 2D writing to 3D free-form micro/nanofabrication. *Small,* **13,** 1600137 (2017)

13. J.-N. Chazalviel, Electrochemical aspects of the generation of ramified metallic electrodeposits. *Phys. Rev. A* **42**, 7355–7367 (1990)

14. R. Bhattacharyya, B. Key, H. Chen, A. S. Best, A. F. Hollenkamp, C. P. Grey, In situ NMR observation of the formation of metallic lithium microstructures in lithium batteries. *Nat. Mater.* **9**, 504–510 (2010)

15. M. Wang, W. J. P. Enckevort, N.-B. Ming, P. Bennema, Formation of a mesh-like electrodeposit induced by electroconvection. *Nature* **367**, 438–441 (1994)

16. V. Fleury, J. H. Kaufman, D. B. Hibbert, Mechanism of a morphology transition in ramified electrochemical growth. *Nature* **367**, 435–438 (1994)

17. V. Fleury, J.-N. Chazalviel, M. Rosso, Theory and experimental evidence of electroconvection around electrochemical deposits. *Phys. Rev. Lett.* **68**, 2492–2495 (1992)

18. M. Wang, S. Zhong, X.-B. Yin, J.-M. Zhu, R.-W. Peng, Y. Wang, K.-Q. Zhang, N.-B. Ming, Nanostructured copper filaments in electrochemical deposition. *Phys. Rev. Lett.* **86**, 3827–3830 (2001)

19. B. Zhang, Y.-Y. Weng, X.-P. Huang, M. Wang, R.-W. Peng, N.-B. Ming, B. Yang, N. Lu, L. Chi, Creating in-plane metallic-nanowire arrays by corner-mediated electrodeposition. *Adv. Mater.* **21**, 3576–3580 (2009)

20. F. Chen, J. Li, F. Yu, D. Zhao, F. Wang, Y. Chen, R.-W. Peng, M. Wang, Construction of 3D metallic nanostructures on an arbitrarily shaped substrate. *Adv. Mater.* **28**, 7193–7199 (2016)

21. K. Peng, D. Jevtics, F. Zhang, S. Sterzl, D. A. Damry, M. U. Rothmann, B. Guilhabert, M. J. Strain, H. H. Tan, L. M. Herz, L. Fu, M. D. Dawson, A. Hurtado, C. Jagadish, M. B. Johnston, Three-dimensional cross-nanowire networks recover full terahertz state. *Science* **368**, 510–513 (2020)

22. Z. Yang, T. Albrow-Owen, H. Cui, J. Alexander-Webber, F. Gu, X. Wang, T.-C. Wu, M. Zhuge, C. Williams, P. Wang, A. V. Zayats, W. Cai, L. Dai, S. Hofmann, M. Overend, L. Tong, Q. Yang, Z. Sun, T. Hasan, Single-nanowire spectrometers. *Science* **365**, 1017–1020 (2019)

23. D. Jacobsson, F. Panciera, J. Tersoff, M. C. Reuter, S. Lehmann, S. Hofmann, K. A. Dick, F. M. Ross, Interface dynamics and crystal phase switching in GaAs nanowires. *Nature* **531**, 317–322 (2016)

24. A. K. Nayak, V. Kumar, T. Ma, P. Werner, E. Pippel, R. Sahoo, F. Damay, U. K. Rößler, C. Felser, S. S. P. Parkin, Magnetic antiskyrmions above room temperature in tetragonal Heusler materials. *Nature* **548**, 561–566 (2017)

25. S. Parkin, S.-H. Yang, Memory on the racetrack. *Nat. Nanotechnol.* **10**, 195–198 (2015)

26. Z. Luo, A. Hrabec, T. P. Dao, G. Sala, S. Finizio, J. Feng, S. Mayr, J. Raabe, P. Gambardella, L. J. Heyderman, Current-driven magnetic domain-wall logic. *Nature* **579**, 214–218 (2020)

27. M. Z. Zhang, Y. Wang, G.-W. Yu, M. Wang, R.-W. Peng, N.-B. Ming, Formation of copper electrodeposits on an untreated insulating substrate. *J. Phys. Condens. Matter* **16**, 695–704 (2004)

28. M. Zhang, S. Lenhert, M. Wang, L. Chi, N. Lu, H. Fuchs, N. Ming, Regular arrays of copper wires formed by template-assisted electrodeposition. *Adv. Mater.* **16**, 409–413 (2004)

29. S. Zhong, Y. Wang, M. Wang, M.-Z. Zhang, X.-B. Yin, R.-W. Peng, N.-B. Ming, Formation of nanostructured copper filaments in electrochemical deposition. *Phys. Rev. E* **67**, 061601 (2003)

30. C. K. Kang, S. M. Lee, I. D. Jung, P. G. Jung, S. J. Hwang, J. S. Ko, The fabrication of patternable silicon nanotips using deep reactive ion etching. *J. Micromech. Microeng.* **18**, 075007 (2008)

31. M. Wang, N.-B. Ming, Alternating morphology transitions in electrochemical deposition. *Phys. Rev. Lett.* **71**, 113–116 (1993)

32. J.-P. Tetienne, T. Hingant, J.-V. Kim, L. H. Diez, J.-P. Adam, K. Garcia, J.-F. Roch, S. Rohart, A. Thiaville, D. Ravelosona, V. Jacques, Nanoscale imaging and control of domain-wall hopping with a nitrogen-vacancy center microscope. *Science* **344**, 1366–1369 (2014)

33. R. Moriya, L. Thomas, M. Hayashi, Y. B. Bazaliy, C. Rettner, S. S. P. Parkin, Probing vortex-core dynamics using current-induced resonant excitation of a trapped domain wall. *Nat. Phys.* **4**, 368–372 (2008)
